# Characteristics of Computed Tomography Images for Patients with Acute Liver Injury Caused by Sepsis under Deep Learning Algorithm

**DOI:** 10.1155/2022/9322196

**Published:** 2022-03-20

**Authors:** Huijun Wang, Qianqian Bao, Donghang Cao, Shujing Dong, Lili Wu

**Affiliations:** ^1^Department of Anesthesiology, Taizhou Hospital of Zhejiang Province Affiliated to Wenzhou Medical University, No. 150 Linhai West Street, Taizhou 317000, Zhejiang, China; ^2^Department of Operation, Taizhou Hospital of Zhejiang Province Affiliated to Wenzhou Medical University, No. 150 Linhai West Street, Taizhou 317000, Zhejiang, China

## Abstract

This study was aimed at exploring the application of image segmentation based on full convolutional neural network (FCN) in liver computed tomography (CT) image segmentation and analyzing the clinical features of acute liver injury caused by sepsis. The Sigmoid function, encoder-decoder, and weighted cross entropy loss function were introduced and optimized based on FCN. The Dice value, precision, recall rate, volume overlap error (VOE), relative volume difference (RVD), and root mean square error (RMSE) values of the optimized algorithms were compared and analyzed. 92 patients with sepsis were selected as the research objects, and they were divided into a nonacute liver injury group (50 cases) and acute liver injury group (42 cases) based on whether they had acute liver injury. The differences in the proportion of patients with different disease histories, the proportion of patients with different infection sites, the number of organ failure, and the time of admission to intensive care unit (ICU) were compared between the two groups. It was found that the optimized window CT image Dice value after preprocessing (0.704 ± 0.06) was significantly higher than the other two methods (*P* < 0.05). The Dice value, precision, and recall rate of the optimized-FCN algorithm were (0.826 ± 0.06), (0.91 ± 0.08), and (0.88 ± 0.09), respectively, which were significantly higher than other algorithms (*P* < 0.05). The VOE, RVD, and RMSE values were (21.19 ± 1.97), (10.45 ± 1.02), and (0.25 ± 0.02), respectively, which were significantly lower than other algorithms (*P* < 0.05). The proportion of patients with a history of drinking in the nonacute liver injury group was lower than that in the acute liver injury group (*P* < 0.05), and the proportion of patients with a history of hypotension was greatly higher than that in the nonacute liver injury group (*P* < 0.01). CT images of sepsis patients with acute liver injury showed that large areas of liver parenchyma mixed with high-density hematoma, the number of organ failures, and the length of stay in ICU were significantly higher than those in the nonacute liver injury group (*P* < 0.05). It showed that the optimization algorithm based on FCN greatly improved the performance of CT image segmentation. Long-term drinking, low blood pressure, number of organ failures, and length of stay in ICU were all related to sepsis and acute liver injury. Conclusion in this study could provide a reference basis for the diagnosis and prognosis of acute liver injury caused by sepsis.

## 1. Introduction

Sepsis is one of the main causes of death in intensive care unit (ICU) patients. According to statistics, there are approximately 18 million new cases each year, with a mortality rate of 2%–40% [1]. As the target organ of sepsis, the liver plays an important role in the occurrence and development of sepsis [2]. Acute liver injury is one of the common acute and critical illnesses caused by sepsis, with a high fatality rate and poor prognosis [3]. However, there are few studies on the clinical characteristics of patients with sepsis complicated by acute liver injury. At present, the diagnosis of sepsis liver injury mainly uses a number of serological indicators to indirectly assess the severity of liver injury [4]. In imaging examination methods, CT examination has significant advantages in evaluating liver trauma or intra-abdominal blood volume. Compared with serological test indicators, CT can reflect the extent of liver parenchymal destruction and accurately determine the degree of liver damage [5]. However, there are abundant organs and blood vessels around the liver, and the boundary between normal tissue and the diseased area is blurred. Manually segmenting the diseased area with CT images is time-consuming and prone to segmentation errors [6].

As a new field of machine learning, deep learning can be established to imitate the human brain for data analysis and learning and has powerful feature learning and model representation capabilities. Convolutional neural network (CNN) has strong representation learning capabilities and has been widely used in speech recognition, language processing, and image processing [7]. CNN has been used in the detection of breast cancer, cell carcinoma and brain lesions, knee cartilage segmentation, brain tumor segmentation, and liver tumor segmentation in medical image processing [8]. However, CNN has the disadvantage of losing the spatial information of the original image in image segmentation [9]. Jiang et al. (2021) [10] pointed out that the full convolutional neural network (FCN) based on CNN can overcome the shortcomings of CNN in the liver segmentation process of CT images and avoid the repeated storage and calculation convolution caused using pixel blocks. However, the image results obtained by the upsampling of the FCN algorithm are smooth and blurred, which is not sensitive to the processing of details in the image [11]. At the same time, some studies pointed out that the relationship between pixels is not fully considered in the image processing process, and the spatial regularization step used in the usual pixel classification-based segmentation methods is ignored, which lacks spatial consistency [12]. Therefore, it needs to be further optimized to overcome the problems of inaccurate segmentation results and lack of spatial consistency of the FCN algorithm.

To sum up, CT shows significant advantages in acute liver injury. The FCN algorithm still has certain limitations in CT image liver segmentation, which needs to be optimized. Moreover, there are few studies on the analysis of CT imaging characteristics of patients with acute liver injury in sepsis. Therefore, the FCN algorithm was optimized to increase its image segmentation performance. It was then applied to liver segmentation of CT images in patients with acute sepsis liver injury, and the clinical characteristics and CT imaging characteristics of patients with acute liver injury in sepsis were discussed, aiming to provide reference basis for the diagnosis of patients with acute liver injury caused by sepsis.

## 2. Materials and Methods

### 2.1. Research Objects and Grouping

In this study, 92 patients with sepsis in hospital from December 2018 to March 2021 were selected as research objects, and all patients underwent CT examination. There were 51 males and 41 females. The age range of patients was 19–86 years, and the average age was 59.94 ± 10.18 years. The inclusion criteria of this study were defined as follows: patients who had performed with CT examination; and patients who were in line with the inclusion criteria of abnormal liver function [13]. Exclusion criteria were defined as follows: patients with past chronic liver damage; patients after liver tumor or partial liver resection; patients with obstructive jaundice and biliary tract disease; and patients with liver damage caused by nonseptic reasons such as drugs. According to whether they had acute liver injury, they were divided into nonacute liver injury group (50 cases) and acute liver injury group (42 cases). This study had been approved by the ethics committee of hospital, and all subjects included in the study had signed the informed consent forms.

### 2.2. CT Examination Method

All patients were instructed to fast for 4–6 hours before examination, and the clinical data such as blood pressure and pulse were measured about 20 minutes before scanning. Plain scan and enhanced examination were performed with CT machine. The patient was placed in supine position and scanned in transverse axial position, with both arms raised and head held, from the xiphoid process to the lower margin of the liver. To reduce the motion artifacts in the image, patients must hold the air with inhalation during scanning or hold the air with calm breathing. Scanning parameter setting was tube voltage of 100 kV, tube current of 100 mA, pitch of 1.0, layer spacing of 7.5 mm, layer thickness of 7 mm, window position of 45–75 HU, and window width of 150–250 HU. According to Becker classification [14], liver injury CT images were classified.

### 2.3. Liver Segmentation Framework Based on FCN

**Figure 1:**
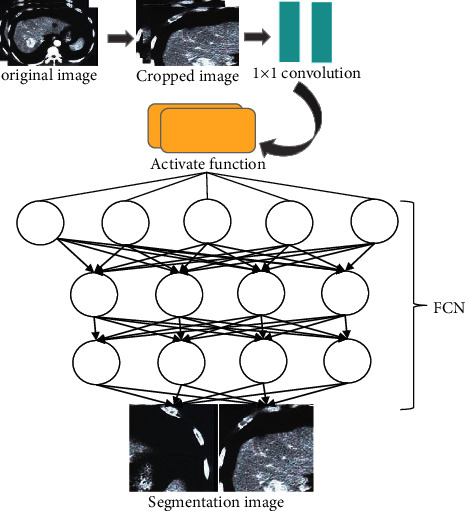
Liver segmentation process based on FCN.

FCN has greatly improved image segmentation accuracy and segmentation speed from traditional CNN [15]. Based on the imprecise segmentation results in the FCN algorithm, the optimization was realized through the CT window and algorithm optimization, and a liver segmentation framework was established, as shown in Figure 1. The initially obtained CT image data set was randomly cropped and then subjected to FCN algorithm for segmentation, and finally the segmented liver CT image was output.

### 2.4. CT Image Preprocessing for Liver Segmentation

Image preprocessing can reduce the interference of a certain tissue or organ from unrelated tissues or organs. The CT value of different tissues and organs in the human body is different, and the CT value of liver tissue is between 50 and 70 HU [16]. For the initial CT image of the liver, it was inputted to the convolutional layer with a convolution kernel size of 1 × 1 for preprocessing, the Sigmoid function was used to activate it, and the preprocessed CT image was finally output. It was assumed that the upper limit of the serial port function of CT image processing was A, and the liver CT image preprocessing module can be expressed as follows:

(1) $$\begin{array}{c} G\left(x\right)=\frac{A}{1+e^{-\left(Cx+b\right)}}. \end{array}$$

In the equation above, *C* and *b* were convolutional layer parameters, and *x* was the CT value of the original CT image. In order to increase the efficiency of network training, the convolutional layer parameters *C* and *b* were optimized in the study. The specific algorithm was as follows:

(2) $$\begin{array}{c} C=\frac{2}{CC}\log\left(\frac{A}{\alpha }-1\right),\\ b=\frac{-2CL}{CC}\log\left(\frac{A}{\alpha }-1\right). \end{array}$$

In the equations above, *CC* was the window width, *CL* was the window level, and *α* was the difference between the upper bound of the function and the right end of the window, which is related to the slope of the Sigmoid function.

### 2.5. Design of Liver CT Image Segmentation Network Based on FCN

The encoder-decoder structure can not only improve the feature expression ability in the image segmentation process, but also avoid the problem of gradient disappearance that may occur during the training process of the deep network [17]. Dense Net can avoid the training error caused by deep network training through the short-circuit connection in the residual module [18]. *l* was assumed to be the current number of layers, and the output of layer *l* can be expressed as follows:

(3) $$\begin{array}{c} x_{l}=B_{l}\left\{x_{0},x_{l},\ldots ,x_{l-1}\right\}. \end{array}$$

In (3), *B*_*l*_{ } represented a combination of operations, and *x*_0_, *x*_*l*_,…, *x*_*l*−1_ was the splicing of the output of all layers before the layer *l*.

**Figure 2:**
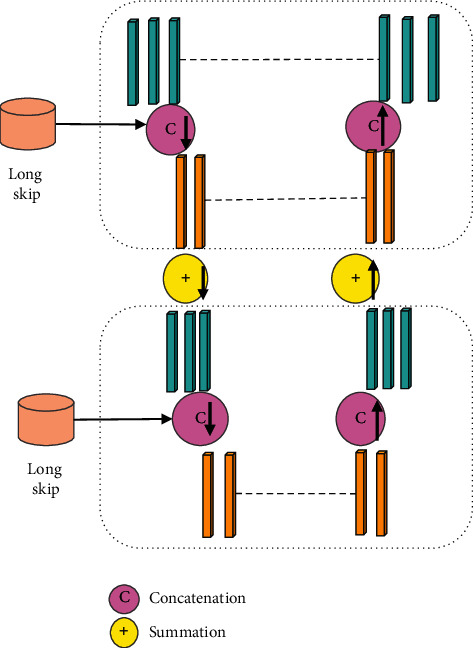
Encoder structure diagram after optimization.

After the input image passed through the encoder, a low-resolution feature representation was produced, and the decoder network structure was related to the final image segmentation result [19]. In this study, the Dense connection among decoder modules was introduced, and the Skip connection in semantic segmentation was introduced to reduce the potential errors caused by the decoder's mesoscale modules, thereby improving the accuracy of segmentation. The structure of the liver CT image processing encoder optimized in this study is shown in Figure 2.

### 2.6. Design of Loss Function of Liver CT Image Segmentation Network Based on FCN

The segmentation evaluation index performance combining cross entropy and Lovász-Softmax's loss function was significantly higher than that of alone [20]. The calculation method of the cross entropy loss function was given as follows:

(4) $$\begin{array}{c} D\left(y,y{\;}^{\prime }\right)=-\frac{1}{N}\sum_{i=1}^{N}\sum_{h=1}^{H}y_{i}^{h}\log\;y_{i}^{\prime h}. \end{array}$$

In (4), *y*_*i*_^*h*^ referred to the binary label of pixel *i* to category *h*, *y*_*i*_^′*h*^ represented the probability value that pixel *i* belongs to category *h*, *N* was the sum of the number of pixels in a batch in the training process, and *H* was the total number of categories.

In liver CT image segmentation, a weighted cross entropy loss function is often used to solve the problem of category imbalance. The calculation method of weighted cross entropy loss function is shown in the following formula, where *ω*_*i*_^*h*^ represented the weight of the category:

(5) $$\begin{array}{c} D\left(y,y{\;}^{\prime }\right)=-\frac{1}{N}\sum_{i=1}^{N}\sum_{h=1}^{H}\omega_{i}^{h}y_{i}^{h}\log\;y_{i}^{\prime h}. \end{array}$$

During the operation of the cross entropy loss function, there was still rough segmentation of the edge area of the image. The Dice coefficient was a commonly used evaluation index for segmented images. The calculation method was given as follows:

(6) $$\begin{array}{c} D\text{ice}\left(A,B\right)=\frac{2\left|A\cap B\right|}{\left|A\right|+\left|B\right|}. \end{array}$$

In the above equation, *A* was the number of pixels in the prediction area, and *B* was the number of pixels in the labeling area. The optimized Dice coefficient can be used as a loss function to improve the rough edge of the segmented image. The Dice loss function can be expressed as

(7) $$\begin{array}{c} \text{Dice}=\frac{2\sum_{i}^{N}P_{i}Q_{i}}{\sum_{i}^{N}P_{i}^{2}+\sum_{i}^{N}Q_{i}^{2}}. \end{array}$$

In (7), *P* represented the predicted probability value of pixel *i*,  _*Q*_^*i*^ was the binary label value of pixel *i*, and *N* referred to the total number of pixels in a batch. The partial derivative of the predicted probability of the Dice coefficient to the j-th pixel and the optimized loss function (sDice) are, respectively, expressed as

(8) $$\begin{array}{c} \frac{\partial D}{\partial P_{j}}=2\left[\frac{Q_{j}\left(\sum_{i}^{N}P_{i}^{2}+\sum_{i}^{N}Q_{i}^{2}\right)-2P_{j}\left(\sum_{i}^{N}P_{i}Q_{i}\right)}{{\left(\sum_{i}^{N}P_{i}^{2}+\sum_{i}^{N}Q_{i}^{2}\right)}^{2}}\right],\\ sD\text{ice}=1-D\text{ice.} \end{array}$$

The Jaccard index is also a measure of the degree of regional overlap [21], and its calculation method was as follows:

(9) $$\begin{array}{c} \text{Jaccard}=\frac{\left|A\cap B\right|}{\left|A\cup B\right|}. \end{array}$$

In semantic segmentation, the Jaccard index can be expressed as

(10) $$\begin{array}{c} J_{c}\left(y,y{\;}^{\prime }\right)=\left|\frac{\left\{y=h\right\}\cap \left\{y{\;}^{\prime }=h\right\}}{\left\{y=h\right\}\cup \left\{y{\;}^{\prime }=h\right\}}\right|. \end{array}$$

In (10), *y* was the actual pixel category label vector and *y'* was the predicted pixel category label vector.

The calculation method of loss function based on Jaccard index was shown in

(11) $$\begin{array}{c} \Delta J_{c}\left(y,y{\;}^{\prime }\right)=1-J_{c}\left(y,y{\;}^{\prime }\right). \end{array}$$

In order to optimize the discrete Jaccard loss in continuous space, it needs to be expanded smoothly. It was assumed that the set of misclassified pixels was *E*_*c*_, which could be expressed as

(12) $$\begin{array}{c} E_{c}\left(y,y{\;}^{\prime }\right)=\left\{y=c,y{\;}^{\prime }\neq c\right\}\cup \left\{y\neq c,y{\;}^{\prime }=c\right\}. \end{array}$$

Jaccard loss could be expressed as

(13) $$\begin{array}{c} \Delta J_{c}:E_{c}\in \left\{0,1\right\}P\mapsto \frac{\left|E_{c}\right|}{\left|\left\{y=c\right\}\cup E_{c}\right|}. \end{array}$$

*H*_*i*_(*x*) was assumed to be the prediction score of the pixel by the segmentation network, and the Hinger loss of the pixel can be expressed as follows:

(14) $$\begin{array}{c} m_{i}=\max\left[1-H_{i}\left(x\right)y_{i},0\right]. \end{array}$$

Then, the final loss can be expressed as

(15) $$\begin{array}{c} \text{Loss}\left(H\right)=\bar{\Delta J_{c}}\left[m\left(H\right)\right]. \end{array}$$

In the equation above, $\bar{\Delta J_{c}}$ referred to the Lovasz expanded item of Δ*J*_*c*_, and *m* was the vector number.

### 2.7. Test Data and Image Evaluation Indicators

In this study, the liver CT images were from 86 patients with liver disease collected. The original liver CT tomographic slice parameters were 1.25 mm ∗  1.25 mm, and the slice spacing was 2 mm. All CT images were randomly divided into a training set (60 cases) and a test set (26 cases). The test environment was defined as follows: computer operating system Ubuntu 16.04 LTS 64 bit system, central processing unit (CPU) : Intel Core I7-2600 3.4 G HZ, memory: DDR 34 GB, hard disk 1 TB. The *Python* 3 was selected as a tool for implementing the DBN model code, and the deep learning framework was PyTorch.

The focus detection mainly used the focus detection precision and recall rate for evaluation, and the calculation methods were as follows:

(16) $$\begin{array}{c} \text{precision}=\frac{TP}{TP+FP}\times 100\%, \end{array}$$

(17) $$\begin{array}{c} \text{recall}=\frac{TP}{TP+FN}\times 100\%. \end{array}$$

In (16) and (17), *TP* represented the number of correctly detected lesions; *FN* represented the number of missed lesions; and *FP* represented the number of wrongly detected lesions.

Image segmentation evaluation indicators mainly used volume overlap error (VOE) and relative volume difference (RVD) for evaluation, and the calculation methods were as follows:

(18) $$\begin{array}{c} \text{VOE}\left(C,D\right)=1-\left|\frac{C\cap D}{C\cup D}\right|,\\ \text{RVD}\left(C,D\right)=\frac{\left|D\right|-\left|C\right|}{\left|C\right|}. \end{array}$$

In the equation above, C referred to the pixel set of the automatically segmented image, and *D* was the pixel set of the manually drawn image.

The root mean square error (RMSE) can be used to evaluate the burden of liver lesions. The calculation method of RMSE was given as follows:

(19) $$\begin{array}{c} \text{RMSE}=\sqrt{\frac{1}{n}\sum_{i=1}^{n}{\left(C_{i}-D_{i}\right)}^{2}}. \end{array}$$

### 2.8. Statistical Analysis

The test data processing was performed using SPSS19.0 statistical software, the measurement data were expressed as mean ± standard deviation ($\bar{x}$ ± *s*), and count data were expressed as percentage (%), using the *χ*^2^ test. *P* < 0.05 indicated that the difference was statistically significant.

## 3. Results and Analysis

### 3.1. Analysis of CT Image Preprocessing Results

**Figure 3:**
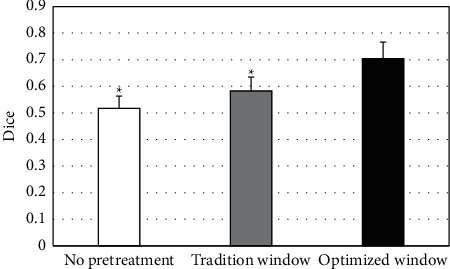
Comparison on the Dice value of CT images under different preprocessing methods.  ^*∗*^ indicates a statistical difference compared with the optimized window method (*P* < 0.05).

The segmented images without pretreatment (no pretreatment), traditional CT window pretreatment (tradition window), and optimized window pretreatment (optimized window) were compared and analyzed. The results were shown in Figure 3. The Dice value of the CT image preprocessed by the optimized window was (0.704 ± 0.06), and the Dice values of the CT image preprocessed by the no pretreatment and tradition window were (0.517 ± 0.05) and (0.583 ± 0.05), respectively. The Dice value of CT image preprocessed by optimized window was significantly higher than the other two methods (*P* < 0.05).

**Figure 4:**
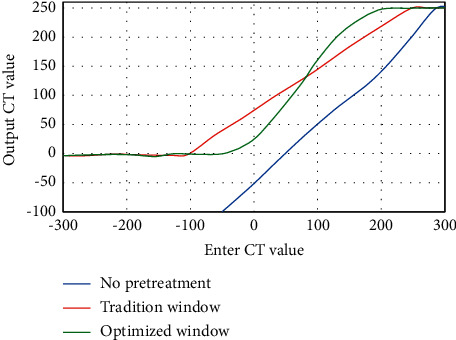
Curves of CT values of CT images under different preprocessing methods.

The output CT values of CT images under different preprocessing methods were compared further, and the results are illustrated in Figure 4. With the continuous increase of the initial CT value, the output CT values of no pretreatment and tradition window both show an obvious linear upward trend; and the output CT value of the optimized window shows an “S” curve with the continuous increase of the initial CT value. When the input CT value was greater than 200 HU, the output CT value no longer changed, showing a stable trend.

### 3.2. Analysis of CT Image Segmentation Effect

**Figure 5:**
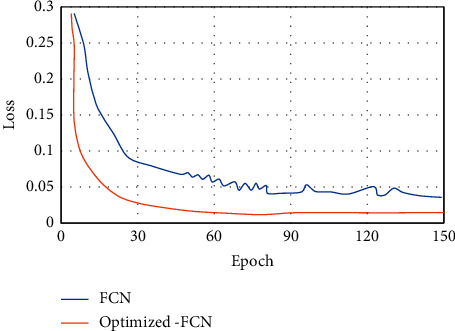
Changes in Loss values on training sets of different algorithms.

The Loss values of the FCN algorithm before optimization and the optimized-FCN algorithm in this study were analyzed and compared on the training set (Figure 5). As the Epoch value continued to increase, the Loss values of the two algorithms showed a tendency to first decrease and then stabilize. Under the same Epoch value, the Loss value of the Optimized-FCN algorithm was lower than that of the FCN algorithm.

**Figure 6:**
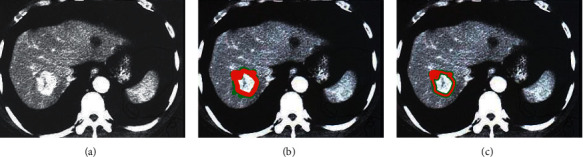
Comparison on segmentation results of liver lesions by different algorithms. (a) Initial CT image; (b) CT image segmented by FCN algorithm; (c) CT image segmented by Optimized-FCN algorithm.

A comparative analysis of the results of segmentation of CT images by the FCN algorithm before optimization and the Optimized-FCN algorithm is shown in Figure 6. It illustrated that, compared with the FCN algorithm, the Optimized-FCN algorithm had a higher degree of fit between the liver CT image lesion segmentation results and the manual segmentation results.

**Figure 7:**
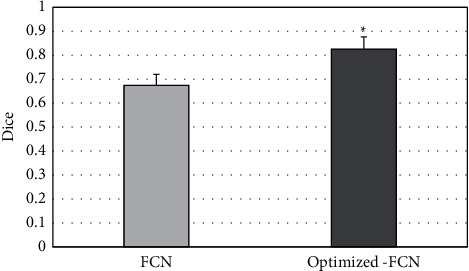
Comparison on Dice value before and after optimization. ^*∗*^ suggests that the difference was statistically obvious (*P* < 0.05).

The Dice value of CT image segmentation between the FCN algorithm before optimization and the Optimized-FCN algorithm was compared.  Figure 7 shows that the Dice values of the FCN algorithm and the optimized-FCN algorithm were (0.675 ± 0.07) and (0.826 ± 0.06), respectively, and the Dice value of the Optimized-FCN algorithm was much higher than that of the FCN algorithm (*P* < 0.05).

### 3.3. Comparative Analysis with Other Segmentation Methods

**Figure 8:**
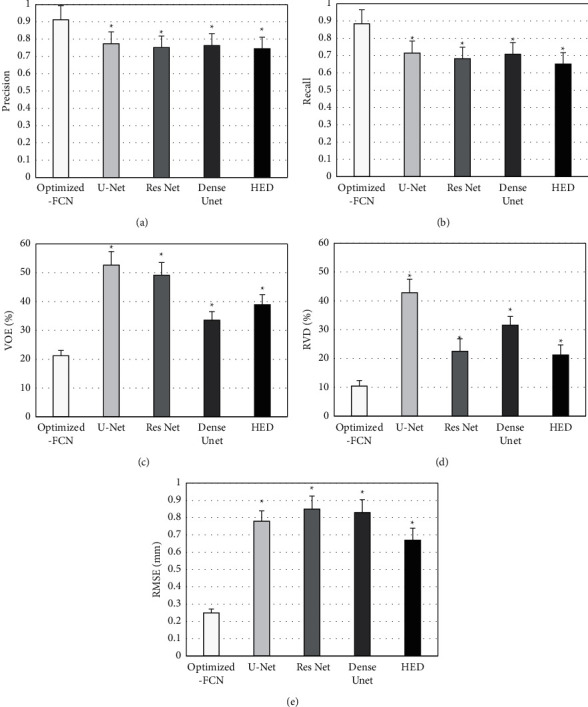
Comparison on image segmentation indexes of different algorithms. (a–e) showed the comparison on precision, recall, VOE, RVD, and RMSE, respectively.  ^*∗*^ means the difference was statistically obvious compared with the Optimized-FCN algorithm (*P* < 0.05).

The results of Optimized-FCN algorithm segmentation of CT images with UNet, Res Net, Dense UNet, and the whole nested edge detection (HED) algorithm were compared (Figure 8). The detection precision, recall, VOE, RVD, and RMSE values of the optimized-FCN algorithm were (0.91 ± 0.08), (0.88 ± 0.09), (21.19 ± 1.97), (10.45 ± 1.02), and (0.25 ± 0.02), respectively. The detection precision and recall rate of Optimized-FCN algorithm were greatly higher than other algorithms (*P* < 0.05), and the values of VOE, RVD, and RMSE were significantly lower than other algorithms (*P* < 0.05).

### 3.4. Comparison of Basic Data

The basic data of the two groups of patients, such as age, gender, smoking history, drinking history, history of hypertension, history of diabetes, and history of low blood pressure, were compared and analyzed (Table 1). There was no significant difference between the two groups of patients in age, gender, the proportion of patients with smoking history, the proportion of hypertension history, and the proportion of diabetes history (*P* > 0.05). The proportion of patients with a history of drinking in the nonacute liver injury group was lower than that in the acute liver injury group (*P* < 0.05), and the proportion of patients with a history of low blood pressure in the acute liver injury group was significantly higher than that in the nonacute liver injury group (*P* < 0.01).

### 3.5. CT Imaging Results of Acute Liver Injury Caused by Sepsis

**Figure 9:**
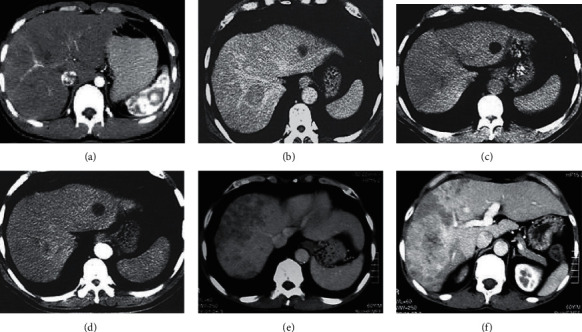
CT imaging results of acute liver injury caused by sepsis. (a–f) The CT images of patients with normal liver, grade I, grade II, grade III, grade IV, and grade V, respectively.

The CT imaging manifestations of patients with acute liver injury under different grades were analyzed, and the results are illustrated in Figure 9. Crescent-shaped high-density hematomas under the liver capsule can be observed in CT imaging of patients with grade I; the left liver lobe of grade II patients had a slightly low-density small hematoma; the right hepatic lobe of grade III patients was irregular and the boundary is blurred; and in the grade IV CT image, the right lobe of the liver can be seen with a patchy high-low density hematoma. The liver parenchyma showed a large mixed high-density hematoma under the grade V CT image, and the liver parenchymal lesions involved the right portal vein.

### 3.6. Clinical Characteristics of Patients with Acute Liver Injury Caused by Sepsis

**Figure 10:**
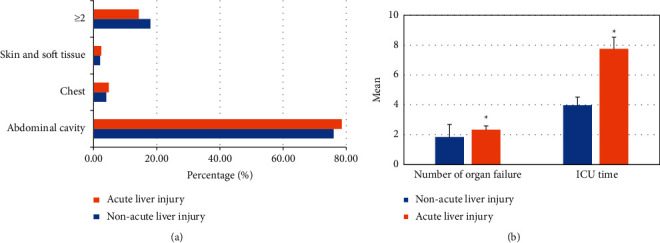
Comparison on infection site, number of organ failures, and length of stay in ICU between the two groups. (a) Comparison on the infection sites; (b) comparison on number of organ failures and length of stay in ICU.  ^*∗*^ suggests that the difference was statistically visible in contrast to the nonacute liver injury group (*P* < 0.05).

The clinical characteristics of the two groups of patients were compared, and the results are revealed in Figure 10. There was no significant difference in the proportion of patients with different infection sites between the two groups (*P* > 0.05). The number of organ failures and length of stay in ICU in the acute liver injury group were much higher than that of the nonacute liver injury group (*P* < 0.05).

## 4. Discussion

Xu et al. (2019) [22] optimized it based on traditional window settings, and the results showed that image segmentation parameters have been improved. This study further optimized it based on the current research results to improve the segmentation performance. The results in the study showed that the Dice value of the CT image preprocessed by the optimized window was obviously higher than the other two methods (*P* < 0.05). It showed that the optimized CT window preprocessing method in this study can improve the performance of liver CT image segmentation. The Loss value of the Optimized-FCN algorithm was lower than that of the FCN algorithm, and the Dice value was higher than that of the FCN algorithm (*P* < 0.05). It showed that, under the same conditions, the loss function value of the optimized-FCN algorithm was lower, which may be related to the selection of weighted cross entropy loss function used in this study. The weighted cross entropy loss function can enable the segmented image to complete the rough segmentation and refined segmentation of the target object in different periods of training [23], improving the segmentation effect and reducing the Loss value. The detection precision and recall rate of the Optimized-FCN algorithm were significantly higher than other algorithms (*P* < 0.05), and the VOE, RVD, and RMSE values were significantly lower than other algorithms (*P* < 0.05). These results showed that the Optimized-FCN algorithm significantly improved the segmentation performance of liver CT images. Alirr (2020) [24] segmented the liver based on a machine learning algorithm and found that the Dice of the segmented CT image was 0.726. Weston et al. (2020) [25] established a liver segmentation method based on the CNN algorithm, and the results showed that the Dice value of this method for segmenting liver CT images was 0.79. Chen et al. (2021) [26] optimized the model based on the FCN algorithm and applied it to liver segmentation. The results showed that the Dice value of this method for segmenting liver CT images was 0.742. In this study, the Dice value of the Optimized-FCN algorithm for segmenting liver CT images was 0.825, which was significantly better than these algorithms.

The typical CT appearance of liver injury is a well-defined low-density change on plain scan, which corresponds to the irradiation field and has nothing to do with liver anatomy [27]. The incidence of acute liver dysfunction in sepsis was about 25%. The results of this study showed that the proportion of patients with a history of drinking and low blood pressure in the nonacute liver injury group was lower than that in the acute liver injury group (*P* < 0.05). The reason was that long-term drinking can cause liver dysfunction, fatty degeneration, and liver compensatory ability to be significantly reduced. When the body develops sepsis, it is accompanied by liver function damage [28]. Insufficient tissue perfusion in patients with severe sepsis leads to hypoxia of weaving cells and ischemia of hepatocytes and ultimately leads to liver damage [29]. The number of organ failures and length of stay in ICU in the acute liver injury group were significantly higher than those in the nonacute liver injury group (*P* < 0.05). The more severe the liver damage, the longer the mechanical ventilation time [30]. Current research results showed that whether there is liver damage in the case of severe infection will affect the level of inflammatory response in the lungs. The incidence of acute lung injury is significantly increased if liver injury is present in sepsis patients, mainly due to the increased inflammatory response in the lung upon liver injury [31]. The results of this study were similar to that.

## 5. Conclusion

In this study, a liver CT image segmentation method based on FCN was established, and the clinical characteristics of acute liver injury caused by sepsis were analyzed. It was found that the optimized-FCN algorithm significantly improved the CT image segmentation performance. Long-term drinking, low blood pressure, number of organ failures, and length of stay in ICU were all related to acute liver injury caused by sepsis. However, there were some shortcomings in this study. It preliminarily analyzed the clinical characteristics of acute liver injury caused by sepsis. In addition, it was a retrospective study, lacking biochemical indicators and other data analysis. In the future work, the clinical features of acute liver injury caused by sepsis would be further analyzed. In conclusion, this work established an effective vertical liver CT image segmentation method based on the FCN algorithm and obtained the relevant influencing factors of sepsis acute liver injury, which provided a reference for the diagnosis and prognosis of sepsis acute liver injury.

## Figures and Tables

**Table 1:** Comparison of basic data of the two groups of patients.

Group	Nonacute liver injury group (*n* = 50)	Acute liver injury group (*n* = 42)	t value or *χ*^2^ value	*P* value
Age (years old)	60.02 ± 5.78	59.96 ± 7.64	1.924	0.227
Males (cases, (%))	28 (56.00)	23 (54.76)	2.427	0.258
History of smoking (cases, (%))	14 (28.00)	12 (28.57)	2.337	0.265
History of drinking (cases, (%))	7 (14.00)	19 (45.24)	4.731	0.018 ^*∗*^
History of hypertension (cases, (%))	16 (32.00)	14 (33.33)	2.019	0.198
History of low blood pressure (cases, (%))	24 (12.00)	38 (90.48)	5.213	0.008 ^*∗*^ ^*∗*^
History of diabetes (cases, (%))	10 (20.00)	8 (19.05)	2.922	0.173

^*∗*^ indicated a statistical difference, *P* < 0.05;  ^*∗∗*^ indicated a highly significant difference, *P* < 0.01.

## Data Availability

The data used to support the findings of this study are available from the corresponding author upon request.
